# The potential applications of artificially modified exosomes derived from mesenchymal stem cells in tumor therapy

**DOI:** 10.3389/fonc.2023.1299384

**Published:** 2024-01-04

**Authors:** Yilin Song, Quanlin Song, Daosheng Hu, Binwen Sun, Mingwei Gao, Xiangnan Liang, Boxin Qu, Lida Suo, Zeli Yin, Liming Wang

**Affiliations:** ^1^ Engineering Research Center for New Materials and Precision Treatment Technology of Malignant Tumors Therapy, The Second Affiliated Hospital of Dalian Medical University, Dalian, China; ^2^ Engineering Technology Research Center for Translational Medicine, The Second Affiliated Hospital of Dalian Medical University, Dalian, China; ^3^ Division of Hepatobiliary and Pancreatic Surgery, Department of General Surgery, The Second Affiliated Hospital of Dalian Medical University, Dalian, China; ^4^ Department of Neurosurgery, The Second Affiliated Hospital of Dalian Medical University, Dalian, China

**Keywords:** mesenchymal stem cells, exosomes, tumor-targeted therapy, anti-cancer, drug delivery

## Abstract

Mesenchymal stem cells (MSCs) have tumor-homing ability and play critical roles in tumor treatment, but their dual influences on tumor progression limit their therapeutic applications. Exosomes derived from MSCs (MSC-exosomes) exhibit great potential in targeted tumor treatment due to their advantages of high stability, low immunogenicity, good biocompatibility, long circulation time and homing characteristics. Furthermore, the artificial modification of MSC-exosomes could amplify their advantages and their inhibitory effect on tumors and could overcome the limit of tumor-promoting effect. In this review, we summarize the latest therapeutic strategies involving artificially modified MSC-exosomes in tumor treatment, including employing these exosomes as nanomaterials to carry noncoding RNAs or their inhibitors and anticancer drugs, and genetic engineering modification of MSC-exosomes. We also discuss the feasibility of utilizing artificially modified MSC-exosomes as an emerging cell-free method for tumor treatment and related challenges.

## 1 Introduction

Mesenchymal stem cells (MSCs) are well-sourced multipotent stem cells with the capacity to differentiate into osteoblasts, chondrocytes, and adipocytes (1). To unify the standard of MSCs, the Mesenchymal and Tissue Stem Cell Committee of the International Society for Cellular Therapy determined the minimum identification criteria for MSCs. First, MSCs must be plastic-adherent when maintained in standard culture conditions. Second, MSCs must express CD105, CD73 and CD90 and lack expression of CD45, CD34, CD14 or CD11b, CD79α or CD19 and HLA-DR surface molecules. Third, MSCs must differentiate into osteoblasts, adipocytes and chondroblasts *in vitro* (2). MSCs have unique advantages in tumor treatment. First, MSCs can be ubiquitously found in many tissues, such as bone marrow (3); adipose (4); umbilical vein (5); umbilical cord blood (6); fetal liver (7); synovial membrane (8); amniotic fluid (9); placenta (10); Wharton’s Jelly (11); human umbilical cord perivascular (12); periodontal ligament (13); dental pulp (14); amnion (15); chorion (15); and human levator veli palatini muscle (16), and are not restricted to tissues of mesodermal origin (**Table 1**). In addition, MSCs can be recruited to injured, inflamed, and hypoxic tissues and the tumor microenvironment, which is termed homing (17–20). Thus, the migratory capacity of MSCs into the tumor microenvironment highlights them as ideal vehicles for tumor-targeted therapy (21). However, we discovered that MSCs have dual tumor-promoting and inhibitory roles in HCC progression (22). Furthermore, according to recent reports, the dual effects of MSCs on tumor progression have been verified in different types of tumors (23–29). In general, MSCs exhibit great potential in tumor treatment, but the dual effects of MSCs on tumor progression have limited their use in tumor therapy. Therefore, exploring a new treatment method for MSCs is of great significance.

**Table 1:** Extensive sources and multilineage differentiation of mesenchymal stem cells.

Cell population	Isolation technique	Morphology	Surface markers	Multilineage differentiation	Reference
BMSCs	Plastic adherent cells isolated from the 1.073 g/ml interface of density gradient centrifugation of bone marrow	Fibroblast-like	Positive: SH2 SH3 Negative: CD34 CD45	Adipogenic differentiation (Oil Red O stain) Osteogenic differentiation (ALP and calcium accumulation) Chondrogenic differentiation (Collagen type II)	Pittenger et al (3)
AMSCs	Plastic-adherent cells isolated from 0.075% collagenase digestion of lipoaspirate	Fibroblast-like	Positive: ASO2 Vimentin Negative: Factor VIII SMA	Adipogenic differentiation (Oil Red O stain) Osteogenic differentiation (ALP and von Kossa stain) Chondrogenic differentiation (Alcian Blue stain and collagen type II) Myogenic differentiation (MyoD1 And myosin heavy chain stain)	Zuk et al (4)
UVMSCs	Plastic-adherent and passage-selective cells isolated by 1% collagenase digestion of umbilical vein endothelial and subendothelial layers	Spindle-shape	Positive: CD29 CD13 CD44 CD49e CD54 CD90 HLA-class1 Negative: CD45 CD14 glycophorin A HLA-DR CD51/61 CD106 CD49d	Adipogenic differentiation (Sudan III stain) Osteogenic differentiation (ALP and von Kossa stain)	Covas et al (5)
UCBMSCs	Plastic adherent cells isolated from the 1.077 g/ml density interface of Ficoll-centrifuged processed umbilical cord blood	Fibroblast-like	Positive: CD29 CD49b CD51 CD44 CD58 CD105 HLA-ABC Negative: CD3 CD7 CD19 CD34 CD45 CD117 CD133 CD135 CD31 CD62 CD33 CD90 HLA-DR	Adipogenic differentiation (Oil Red O stain) Osteogenic differentiation (Alizarin red, von Kossa stain and OPN, OCN, type I collagen expression) Chondrogenic differentiation (Safranin-O stain and collagen type II) Neurogenic differentiation (GFAP, MAP-2 expression) Hepatogenic differentiation (Take up LDL and albumin expression)	Lee et al (6)
UCBMSCs	Plastic adherent cells isolated from the 1.077 g/ml density interface of Ficoll-centrifuged processed umbilical cord blood	Fibroblast-like	Positive: CD29 CD49b CD51 CD44 CD58 CD105 HLA-ABC Negative: CD3 CD7 CD19 CD34 CD45 CD117 CD133 CD135 CD31 CD62 CD33 CD90 HLA-DR	Adipogenic differentiation (Oil Red O stain) Osteogenic differentiation (Alizarin red, von Kossa stain and OPN, OCN, type I collagen expression) Chondrogenic differentiation (Safranin-O stain and collagen type II) Neurogenic differentiation (GFAP, MAP-2 expression) Hepatogenic differentiation (Take up LDL and albumin expression)	Lee et al (6)
fMSCs	Plastic adherent cells isolated from the 1.073 g/ml density interface of Percoll-centrifuged fetal livers	Spindle-shaped Fibroblast-like	Positive: CD29 CD44 CD166 CD105(SH2) SH3 SH4 Negative: CD14 CD34 CD45	Adipogenic differentiation (Oil Red O stain) Osteogenic differentiation (Von Kossa stain) Chondrogenic differentiation (Alcian green and HE stain)	Gotherstrom et al (7)
SMMSCs	Plastic-adherent cells culture isolated by 0.2% collagenase digestion of synovium specimens	Fibroblast-like	Positive: β1 β5 αv integrin CD44 VCAM-1 et al Negative: CD45 CD3d CD20 CD14 FLK-1 et al	Adipogenic differentiation (Oil Red O stain) Osteogenic differentiation (Alizarin red and ALP stain) Chondrogenic differentiation (Toluidine blue/safranin-O stain and collagen II)	De Bari et al (8)
AFMSCs	Plastic-adherent cell culture	Fibroblast-like	Positive: SH2 SH3 SH4 CD29 CD44 HLA-ABC CD90 CD105 Negative: CD10 CD11b CD14 CD34 CD117 HLA-DR/DP/DQ EMA	Adipogenic differentiation (Oil Red O stain) Osteogenic differentiation (ALP and von Kossa stain) Neurogenic differentiation (Neuron-specific class III β-tubulin)	Tsai et al (9)
PMSCs	Plastic adherent cells isolated from minced, hemoclastic and trypsinized placenta	Fibroblast-like	Positive: CD54 CD29 CD44 Negative: CD45 CD31 CD133	Adipogenic differentiation (Oil Red O stain) Osteogenic differentiation (Alizarin Red S stain)	Fukuchi et al (10)
Wharton’s Jelly MSCs	Collagenase and trypsin digestion of Wharton’s Jelly primary cell culture	Fibroblast-like	Positive: CD44 CD105 CD29 CD51 SH2 SH3 Negative: CD34 CD45	Adipogenic differentiation (Oil Red O stain and PPARγ2 expression) Osteogenic differentiation (ALP and von Kossa stain) Chondrogenic differentiation (Alcian Blue stain and collagen type II) Cardiogenic differentiation (Cardiac troponin I or F-actin)	Wang et al (11)
HUCPV-MSCs	Plastic adherent cells isolated from 1 mg/ml collagenase processed umbilical cord vessel and cultured after magnetic bead conjugated-CD45 depletion protocol	Fibroblastic morphology	Positive: CD105(SH2) CD44 CD73(SH3) CD90 (Thy-1) Negative: CD45 CD34, CD235a (glycophorin A) CD106(VCAM1) CD123 (IL3) SSEA-4 HLA-G Oct4 HLA-DR/DP/DQ (MHCII)	Osteogenic differentiation (ALP and von Kossa stain)	Sarugaser et al (12)
PDLSCs	Plastic-adherent cells culture isolated from periodontal ligament tissues digested by 3 mg/mL collagenase type I and 4 mg/mL dispase	Fibroblast-like	Positive: STRO-1 CD146/MUC18	Adipogenic differentiation (Oil Red O stain) Osteogenic differentiation (Alizarin Red S stain)	Seo et al (13)
DP-MSCs	Plastic-adherent cell culture	Spindle-shaped Fibroblast-like	Positive: SH2 SH3 SH4 Negative: CD14 CD34 CD45	Adipogenic differentiation (Oil Red O stain) Osteogenic differentiation (ALP and von Kossa stain)	Pierdomenico et al (14)
Amnion MSCs	Plastic adherent cells isolated from amnion	Fibroblast-like	Positive: CD105, CD90, CD73 Negative: CD11b, CD34, CD45, CD79a, HLA-DR	Adipogenic differentiation (Oil Red O stain) Osteogenic differentiation (silver nitrate) Chondrogenesis differentiation (Alcian blue)	Kwon A et al (15)
CMSCs	Plastic-adherent cells isolated from amnion	Fibroblast-like	Positive: CD105, CD90, CD73 Negative : CD11b,CD34, CD45,CD79a, HLA-DR	Adipogenic differentiation (Oil Red O stain) Osteogenic differentiation (silver nitrate) Chondrogenesis differentiation (Alcian blue) Cardiomyocyte differentiation (Cardiac troponin I or F-actin)	Kwon A et al (15)
Human levator veli palatini muscle MSCs	Plastic-adherent cells isolated from human levator veli palatini muscle	Fibroblast-like	Positive: CD29, CD44, CD73, CD90, CD105 Negative: CD34, CD45, CD31	Adipogenic differentiation (Oil Red O stain) Chondrogenic differentiation (toluidine blue staining) Osteogenic differentiation (Alizarin Red S stain)	Bueno DF et al (16)

BMSCs, bone marrow-derived mesenchymal stem cells; AMSCs, adipose-derived mesenchymal stem cells; UVMSCs, umbilical vein-derived mesenchymal stem cells; UCBMSCs, umbilical cord blood-derived mesenchymal stem cells; OPN, osteopontin; OCN, osteocalcin; LDL, low-density lipoprotein; fMSCs, fetal liver-derived mesenchymal stromal cells; SMMSCs, synovial membrane-derived mesenchymal stem cells; AFMSCs, amniotic fluid-derived mesenchymal stem cells; PMSCs, placenta-derived mesenchymal stem cells; HUCPV-MSCs, human umbilical cord perivascular-derived mesenchymal stem cells; PDLSCs, periodontal ligament stem cells; DP-MSCs, dental pulp-derived mesenchymal stem cells; Amnion MSCs, amnion-derived mesenchymal stem cells; CMSCs, chorion-derived mesenchymal stem cells; Human levator veli palatini muscle MSCs, human levator veli palatini muscle-derived mesenchymal stem cells.

Currently, there is a growing consensus regarding the application value of exosomes in tumor-targeted therapy. Exosomes are nanoparticles approximately 40–200 nm in diameter that are saucer shaped under an electron microscope and have special expression patterns represented by markers such as CD9, CD63, CD81 and TSG101 (30, 31). Exosomes released by living cells can carry bioactive substances, such as cytokines, RNA, DNA, and proteins, and are defined as new tools for tumor treatment due to their ability to transfer bioactive substances or anticancer agents into target cells (32–38). Notably, exosomes derived from MSCs (MSC-exosomes) have the advantages of high stability, low immunogenicity, good biocompatibility, and a long period of circulation as well as a homing mechanism similar to MSCs, so MSC-exosomes could be appropriate carriers to transfer bioactive molecules or therapeutic materials to influence the extracellular environment and cancer biology to treat tumors in a safe way (33). In recent studies, MSC-exosomes have been found to have great potential for tumor therapy (39–43). However, the characteristics of MSC-exosomes in promoting the progression of tumors, including promoting tumor growth (44–46), metastasis (45, 47), immunosuppression (48) drug resistance (49) and dormancy in cancer cells (50), limit their application in tumor therapy. We need to explore measures to address restrictions. An increasing number of researchers have proven that modification of MSC-exosomes could overcome these limitations and amplify the advantages of MSC-exosomes in tumor treatment (51–57). Above all, artificially modified MSC-exosomes may eliminate the influence of MSC-exosomes on tumor progression and provide an effective therapy for tumors.

**Figure 1:**
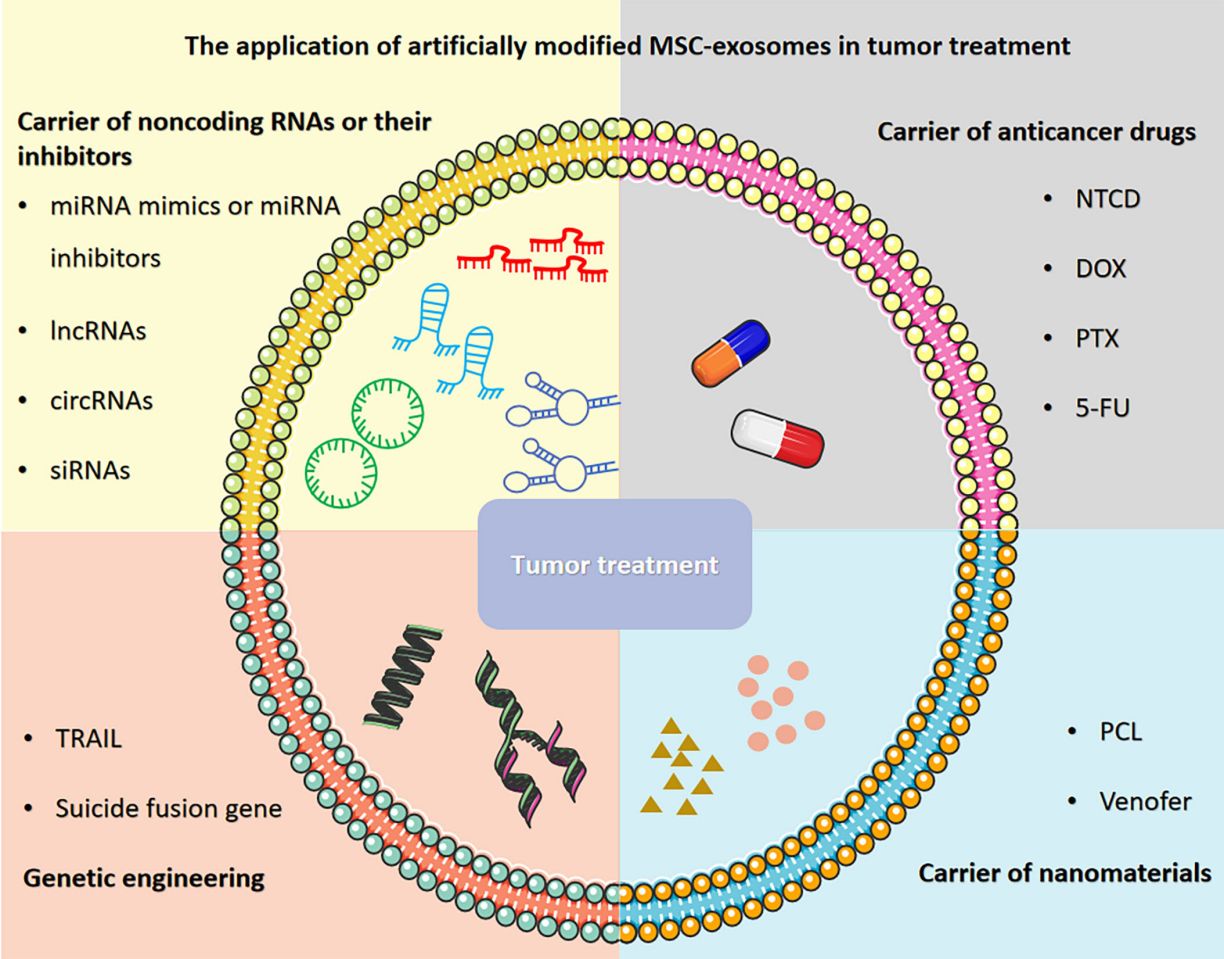
The application of artificially modified MSC-exosomes in tumor treatment includes carriers of noncoding RNAs or their inhibitors, carriers of anticancer drugs, carriers of nanomaterials and genetic engineering. The figure was partly generated using Servier Medical Art, provided by Servier, licensed under a Creative Commons Attribution 3.0 unported license.

Herein, we provide an overview of recent research progress and describe the application of artificially modified MSC-exosomes in tumor treatment, including carriers of noncoding RNAs or their inhibitors, carriers of anticancer drugs, carriers of nanomaterials and genetic engineering (**Figure 1**). We also discuss the prospects and challenges regarding the application of MSC-exosomes in tumor treatment to connect the gaps in knowledge and highlight therapeutic opportunities. Furthermore, we also explore the clinical prospects of applying artificially modified MSC-exosomes in tumor treatment and address the feasibility, safety and challenges of artificially modified MSC-exosomes and provide potential solutions.

## 2 MSC-exosomes carrying noncoding RNAs or their inhibitors contribute to tumor treatment

Noncoding RNAs (ncRNAs) are genes that are not translated to proteins. They can be divided into different categories according to the size, biogenesis and protein partners of ncRNAs and mainly include microRNAs (miRNAs), small interfering RNAs (siRNAs), PIWI-interacting RNAs (piRNAs), long noncoding RNAs (lncRNAs) and circular RNAs (circRNAs) (58–60). Because ncRNAs cannot code for proteins, it is generally believed that ncRNAs have no function, so ncRNAs are referred to as “junk RNAs” (61). However, recent discoveries have revealed that ncRNAs can affect the expression of other genes through a variety of mechanisms; for example, the binding of miRNAs to the untranslated regions of oligonucleotides could block the effects of oligonucleotides and inhibit gene expression (62, 63). Recent studies have shown that ncRNAs are involved in the progression of different types of diseases, such as cardiac disease (64), neurological diseases (65), bipolar disorders (66), and osteoarthritis (67). Moreover, abundant evidence has shown that ncRNAs are closely related to the occurrence, development, invasion, metastasis and drug resistance of tumors (60). Based on the excellent characteristics of ncRNAs in tumor progression, experiments on the application of ncRNAs in tumor therapy have been widely carried out, which show that changing the expression levels of ncRNAs by transferring mimics and inhibitors of ncRNAs can affect the progression of tumors, thus achieving therapeutic effects (68–72). In recent reports, transferring mimics and inhibitors of ncRNAs into tumor cells through MSC-exosomes has been a very promising strategy in tumor treatment. Here, we summarize the application of MSC-exosomes carrying noncoding RNAs or their inhibitors in tumor treatment.

### 2.1 Effects of MSC-exosomes with miRNA mimics or miRNA inhibitors on tumor treatment

MiRNAs are a class of 21-23 nucleotide-long, single-stranded noncoding RNAs that regulate posttranscriptional gene expression by inhibiting the process of translation and by promoting mRNA degradation (73–76). It has been reported that miRNAs play essential roles in tumor development by affecting the proliferation, apoptosis, drug resistance, epithelial mesenchymal transition (EMT), cell cycle and metastasis of tumor cells (35, 77–91). Due to the indispensable function of miRNAs in tumor progression, miRNAs could be one of the potentially effective methods for targeted treatment of tumors. MiRNA mimics are synthesized through chemical synthesis methods to simulate endogenous miRNAs in organisms and could enhance the function of endogenous miRNAs (75, 92). In contrast, miRNA inhibitors are a type of inhibitor that are chemically modified and can prevent complementary pairing between miRNAs and their target gene mRNAs, and inhibit the function of miRNAs by strongly competitive binding with mature miRNAs (75). Recent studies reported that MSC-exosomes with miRNA mimics or miRNA inhibitors could be internalized by tumor cells and change the amounts of miRNAs in tumor cells to regulate the corresponding pathways to influence tumor progression and play an important role in tumor therapy (79, 80, 93–95). The loading of MSC-exosomes with miRNA mimics or miRNA inhibitors may be a safe, effective and promising strategy for tumor treatment.

#### 2.1.1 Preparation of MSC-exosomes loaded with miRNA mimics or miRNA inhibitors

At present, the preparation of MSC-exosomes loaded with miRNA mimics or miRNA inhibitors involves two main approaches. First, MSC-exosomes can be modified directly, and the methods currently used for directly modifying MSC-exosomes include electroporation (96–98) and transfection through exosome transfection reagents (99). It has been reported that miR-3182 mimics, miR-142-3p mimics and miR-142-3p inhibitors can be transferred into MSC-exosomes by electroporation, and the membrane of MSC-exosomes can form transient pores through brief electrical pulses, which allow them to enter MSC-exosomes (96–98). Furthermore, miRNA mimics or miRNA inhibitors could be loaded into MSC-exosomes via exosome transfection solution. Ding Y et al. obtained MSC-exosomes with miR-145-5p mimics by simply mixing MSC-exosomes, miR-145-5p mimics and exosome transfection solution (99). Indirect modification of MSC-exosomes has also been widely used, the first step of which is to obtain MSCs containing miRNA mimics or miRNA inhibitors through two main transfection methods: lipofectamine transfection (51, 81, 84) and lentivirus transduction (100, 101). Recent reports revealed that miR-424 mimics, miR-424-5p mimics and miR-139-5p mimics could be transferred into MSCs through Lipofectamine transfection, and this method shows high transfection efficiency, but Lipofectamine exhibits certain cytotoxicity (51, 81, 84). Sheykhhasan M and Yan T et al. found that miR-145 mimics and miR-512-5p mimics could be transferred into MSCs through lentivirus transduction, which resulted in better stability; however, the load of lentivirus was limited. Moreover, researchers have also obtained MSCs containing miRNA mimics or miRNA inhibitors by coculturing MSCs with miRNA mimics or miRNA inhibitors. Du L et al. transferred miR-21-5p mimics and miR-21-5p inhibitor into MSCs by coculturing miR-21-5p mimics and miR-21-5p inhibitor with MSCs (102). Then, MSC-exosomes could be isolated from MSCs modified by miRNA mimics or miRNA inhibitors. Although several approaches and methods that produce specific MSC-exosomes have been established, the efficiency of these approaches and methods has not been compared. Further studies are needed to identify the most efficient method of generating specific MSC-exosomes to facilitate the application of these structures in tumor treatment.

#### 2.1.2 Application of MSC-exosomes containing miRNA mimics or miRNA inhibitors in tumor therapy

It is widely known that miRNA expression is altered in tumors, and the differential expression of miRNAs influences their effects on tumor growth, highlighting their potential in tumor therapy (103). Due to the dual effect of miRNAs in tumor progression, two types of approaches can be used to adjust the expression level of miRNAs for tumor therapy: suppressing the expression of miRNAs that promote the biological processes of tumors by transferring miRNA inhibitors to tumor cells and enhancing the expression of miRNAs that inhibit the biological processes of tumors by transferring miRNAs to tumor cells. Compared with transferring miRNA mimics or miRNA inhibitors directly into tumor cells, transferring miRNA mimics or miRNA inhibitors via MSC-exosomes has many advantages. First, MSC-exosomes could enhance the inhibitory effect of miRNA mimics or miRNA inhibitors. Shimbo et al. compared the delivery efficiency of miR-143 in osteosarcoma cells subjected to MSC-exosome treatment or lipofection and found that the efficiency of MSC-exosome delivery was lower than that of lipofection, but the inhibitory effect on cell migration was similar in the migration assay (85). Furthermore, MSC-exosomes have excellent tumor cell-targeting ability and biosafety. Due to the advantages of MSC-exosomes, these structures could be exploited as a potentially efficient tool for transferring miRNA mimics or miRNA inhibitors to facilitate tumor treatment.

Considering that some miRNAs can inhibit the progression of cancers and that the expression of miRNAs decreases in some cancer tissues and cells, artificially increasing the expression of miRNAs in cancer cells through MSC-exosomes has a potential inhibitory effect on tumor progression. Chen Z et al. found that miR-6785-5p was downregulated and inhibin A (INHBA) was upregulated in gastric cancer tissues and cells, and miRNA-6785-5p was targeted by INHBA. The malignant development of gastric cancer cells could be restricted by elevating the expression of miR-6785-5p or inhibiting INHBA. MSCs that transferred miR-6785-5p mimics could release exosomes enriched with miR-6785-5p into gastric cancer cells to increase the expression of miR-6785-5p in gastric cancer cells and then reduce the expression of INHBA to suppress angiogenesis and metastasis in gastric cancer (82). There is a lack of evidence regarding the *in vivo* effects of this novel treatment modality. However, the hypothesis that MSC-exosomes with miRNA mimics could inhibit the progression of cancer *in vivo* could be proven through Jia Y’s experiment. They injected MSC-exosomes with and without miR-139-5p mimics into subcutaneous xenograft model mice via the tail vein and found that the presence of miR-139-5p in exosomes reduced the volume and weight of tumors in nude mice. miR-139-5p was downregulated and PCR1 was upregulated in tumor tissues. Overall, MSC-exosomes with miR-139-5p mimics restrain tumorigenesis in bladder cancer by targeting PCR1 *in vivo* (84). In addition to the above two studies, a variety of studies have reported the effect of MSC-exosomes carrying miRNAs mimics on the progression of tumors; these miRNA mimics cargoes include miR-16-5p (104), miR-4461 (105), miR-7 (106), miR-199a (107), miR-133b (108), miR-29a-3p (35), miR-144 (109), miR-193a (110), miR-143 (85), miR-206 (111), miR-1231 (32), miR-152 (112), miR-205 (113), miR-101-3p (114), miR-124a (115), miR-584-5p (116), miR-512-5p (100), miR-204 (79), miR-424-5p (51), miR-122 (117), miR-199a (118), miR-145 (101), miR-181a (80), miR-375 (86), miR-6785-5p (82), miR-139-5p (84), miR-3940-5p (119), miR-21-5p (102), miR-124 (120), miR-34a (52), miR-3182 (98), miR-145-5p (99), miRNA-222-3p (121) (**Table 2**).

**Table 2:** Effect of MSC-exosomes enriched with miRNAs on tumor progression.

Indirect modification of MSCs-exosomes										
Exosome source		miRNAs	Methods of loading		Tumor type		Effect on tumor(*in vitro*)	Effect on tumor(*in vivo*)	Mechanism	Reference
BMSCs		miR-16-5p	Lipofectamine transfection		Colorectal cancer		Inhibition of proliferation, migration, and invasion	Repression of tumor growth	Downregulation of ITGA2	Xu, Y et al (104)
BMSCs		miR-4461	Lipofectamine transfection		Colorectal cancer		Repression of proliferation, migration and invasion		Downregulation of COPB2	Chen, H. L et al (105)
BMSCs		miR-7	Lipofectamine transfection		Glioblastoma		Increase sensitivity to TRAIL-induced apoptosis		Targeting of XIAP	Zhang, X et al (106)
BMSCs		miR-199a	Lipofectamine transfection		Glioma		Suppression of proliferation, invasion and migration	Inhibition of tumor growth	Downregulation of AGAP2	Yu L et al. (107)
BMSCs		miR-133b	Lipofectamine transfection		Glioma		Repression of proliferation, invasion, and migration	Repression of tumor growth	Inhibition of EZH2 and the Wnt/β-catenin signaling pathway	Xu, H et al (108)
BMSCs		miR-29a-3p	Lipofectamine transfection		Glioma		Inhibition of migration and VM	Inhibition of migration and VM	Targeting of ROBO1	Zhang, Z et al (35)
BMSCs		miR-144	Lipofectamine transfection		Non-small cell lung cancer		Repression of proliferation, colony formation, and S-phase arrest	Repression of tumor growth	Downregulation of CCNE1 and CCNE2	Liang, Y et al (109)
BMSCs		miR-193a	Lipofectamine transfection		Non-small cell lung cancer		Suppression of colony formation, invasion, proliferation, migration and promotion of apoptosis of NSCLC cells and drug-resistant cells	Repression of tumor growth	Downregulation of LRRC1	Wu, H (110)
BMSCs		miR-143	Lipofectamine transfection		Osteosarcoma		Inhibition of migration			Shimbo, K et al (85)
BMSCs		miR-206	Lipofectamine transfection		Osteosarcoma		Inhibition of proliferation, migration and invasion and induction of apoptosis	Suppression of osteosarcoma growth and metastasis	Downregulation of TRA2B	Zhang, H (111)
BMSCs		miR-1231	Lipofectamine transfection		Pancreatic cancer		Inhibition of proliferation, migration, invasion, and adhesion to the matrix	Repression of tumor growth		Shang, S et al (32)
BMSCs		miR-152	Lipofectamine transfection		Thyroid carcinoma		Inhibition of proliferation, migration and invasion		Binding with DPP4	Tang, M et al (112)
BMSCs		miR-205	Lentivirus transduction		Prostate cancer		Repression of proliferation, invasion, and migration and enhancement of apoptosis	Repression of tumor growth	Inhibition of RHPN2	Jiang, S et al (113)
BMSCs		miR-101-3p	Lentivirus transduction		Oral cancer		Suppression of proliferation, invasion, and migration	Suppression of tumor growth	Downregulation of COL10A1	Xie, C et al (114)
BMSCs		miR-124a	Lentivirus transduction		gliomas		Inhibition of proliferation and clonogenicity	Increased survival	Inhibition of FOXA2 and alteration of GSC lipid metabolism	Lang, F. M et al (115)
BMSCs		miR-584-5p	Lentivirus transduction		Glioma		Inhibition of proliferation and migration	Inhibition of tumor growth	Targeting of CYP2J2/AKT and MAPK pathways	Kim, R et al (116)
BMSCs		miR-512-5p	Lentivirus transduction		Glioblastoma		Inhibition of proliferation and induction of cell cycle arrest	Inhibition of growth and prolongation of survival	Suppression of JAG1	Yan, T et al (100)
BMSCs		miR-204	Transfection		Non-small cell lung cancer		Impairment of cell migration, invasion and EMT		Inhibition of KLF7 and the AKT/HIF-1α pathway	Liu, X. N et al (79)
AMSCs		miR-424-5p	Lipofectamine transfection		Triple negative breast cancer		Increased secretion of proinflammatory cytokines, decreased secretion of anti-inflammatory cytokines and increased apoptosis	Slowed tumor growth	Downregulation of PD-L1	Zhou, Y et al (51)
AMSCs		miR-122	Lipofectamine transfection		Hepatocellular carcinoma		Resensitization to chemotherapeutic agents	Increased antitumor efficacy of sorafenib	Downregulation of CCNG1, ADAM10 and IGF1R	Lou, G et al (117)
AMSCs		miR-199a	Lentivirus transduction		Hepatocellular carcinoma		Sensitization of HCC cells to doxorubicin	Increased effect of Dox against HCC	Inhibition of the mTOR pathway	Lou, G et al (118)
AMSCs		miR-145	Lentivirus transduction		Breast cancer		Induction of apoptosis and inhibition of metastasis		Downregulation of ROCK1, ERBB2 and MMP9	Sheykhhasan, M et al (101)
UCMSCs		miR-181a	Lipofectamine transfection		Nasopharyngeal carcinoma		Restriction of cell growth	Inhibition of tumor growth	Downregulation of KDM5C	Liu, J et al (80)
UCMSCs		miR-375	Lipofectamine transfection		Esophageal squamous cell carcinoma		Suppression of proliferation, invasion, migration, tumorsphere formation and promotion of apoptosis	Inhibition of tumor growth	Downregulation of ENAH	He, Z et al (86)
UCMSCs		miR-6785-5p	Lipofectamine transfection		Gastric cancer		Suppression of angiogenesis and metastasis		Downregulation of INHBA	Chen, Z (82)
UCMSCs		miR-139-5p	Lipofectamine transfection		Bladder cancer		Inhibition of proliferation, migration, and invasion potentials	Repression of tumor growth	Downregulation of PRC1	Jia, Y et al (84)
UCMSCs		miR-3940-5p	Coculturing		Colorectal cancer		Inhibition of EMT and invasion	Suppression of tumor metastasis and growth	Targeting of Integrin α6	Li T et al. (119)
UCMSCs		miR-21−5p	Coculturing		Breast cancer		Inhibition of migration and invasion		Downregulation of ZNF367	Du, L et al (102)
Wharton’s Jelly MSCs		miR-124	Lipofectamine transfection		Glioblastoma multiforme		Enhancement of the chemosensitivity to temozolomide and decreased migration		Downregulation of CDK6	Sharif, S et al (120)
DP-MSCs		miR-34a	Lentivirus transduction		Triple-negative breast cancer		Repression of proliferation, invasion, and migration			Vakhshiteh, F et al (52)
Direct modification of MSCs-exosomes										
UCMSCs		miR-3182	Electroporation		Triple-Negative Breast Cancer		Inhibition of proliferation and migration and induction of apoptosis		Downregulation of mTOR and S6KB1	Khazaei-Poul, Y et al (98)
UCMSCs		miR-145-5p	Exosome transfection solution		Pancreatic ductal adenocarcinoma		Inhibition of proliferation, invasion and increased apoptosis and cell cycle arrest	Inhibition of tumor growth	Downregulation of Smad3	Ding, Y (99)
BMSCs		miR-222-3p	Incubation		Acute Myeloid Leukemia		Increased Th1/Th2 Ratio and promoted Apoptosis of Acute Myeloid Leukemia Cells		Downregulation of IRF2	Yuan, Y et al (121)

BMSCs, bone marrow-derived mesenchymal stem cells; AMSCs, adipose-derived mesenchymal stem cells; UCMSCs, umbilical cord-derived mesenchymal stem cells; DP-MSCs, dental pulp derived mesenchymal stem cells.

miRNAs can also promote the progression of cancer; the expression of miRNAs is increased in some cancer tissues and cells, and the progression of cancer could be inhibited by artificially decreasing the expression of miRNAs in cancer cells. Zhang N et al. found that MSC-derived exosomes with a miR-424 inhibitor could reduce the expression of miR-424 in colorectal cancer cells and inhibit the progression of tumors by upregulating TGFBR3 (81); Naseri Z et al. discovered that MSC-derived exosomes carrying a miR-142-3p inhibitor could reduce the tumorigenicity of breast cancer *in vitro* and *in vivo*, highlighting the clinical potential of this method for cancer treatment (97). Furthermore, Naseri Z et al. utilized MSC-exosomes to deliver a miR-142-3p inhibitor that was modified by locked nucleic acid molecules into breast cancer stem cells to reduce the tumorigenicity of breast cancer stem cells (96). In general, the processes of proliferation, apoptosis, drug resistance and EMT could be inhibited by reducing the expression of tumor-promoting miRNAs via MSC-exosomes containing miRNA inhibitors to achieve the effect of tumor treatment.

Overall, MSC-exosomes containing miRNA mimics or miRNA inhibitors have great potential in tumor treatment, but the effects of different miRNAs on different tumors are still unclear. Moreover, there are still a series of questions to address regarding the application of MSC-exosomes containing miRNA mimics or miRNA inhibitors in tumor treatment; these questions pertain to the selection of MSC types, the manner of exosome extraction, and the selection of a safe dose of exosomes *in vivo*.

### 2.2 Effects of MSC-exosomes containing lncRNAs on tumors

LncRNAs are an important class of noncoding RNA molecules; they are transcripts longer than 200 nucleotides and participate in a range of biological functions by regulating the expression and functions of genes at the transcriptional, translational, and posttranslational levels (126, 127). Although lncRNAs cannot code for proteins, it has been reported that lncRNAs are involved in some aspects of oncology, including tumorigenesis, metastasis, chemoresistance, cancer stem cell function and EMT (127–130).

Because lncRNAs play an important role in tumor formation and progression and dysregulated lncRNAs exhibit distinct biological functions to promote or suppress tumor growth, regulating their expression has become a potential strategy for tumor treatment. Similar to the approach involving miRNAs, lncRNAs could be employed to inhibit the biological processes of tumors by transferring lncRNAs to tumor cells to enhance their expression for tumor treatment. Moreover, because of the advantages of MSC-exosomes over other transfer strategies, MSC-exosomes containing lncRNAs may become a new mode of tumor treatment (**Table 3**). Hao SC et al. found that the expression of lncRNA PTENP1 was apparently lower in glioma samples than in normal tissues, that lncRNA PTENP1 could inhibit the proliferation of glioma, and that lncRNA PTENP1 could be packaged into MSC-exosomes and transferred to glioma cells and then stabilize PTENP1 by competitively binding miR-10a-5p to improve the antitumor capacity (122).

**Table 3:** Effect of MSC-exosomes enriched with other types of noncoding RNAs (lncRNAs, circRNAs, siRNA) on tumor progression.

Exosome source	Noncoding RNAs	Methods of loading	Tumor type	Effect on tumor (*in vitro*)	Effect on tumor (*in vivo*)	Mechanism	Reference
UCMSCs	lncRNA PTENP1	Lipofectamine transfection	Glioma	Inhibition of proliferation		Regulation of miR-10a-5p/PTEN signaling	Hao, S. C. et al (122)
BMSCs	circ_0030167	Lipofectamine transfection	Pancreatic cancer	Inhibition of the invasion, migration, proliferation and stemness of pancreatic cancer cells	Inhibition of tumor growth	Regulation of miR-338-5p, enhanced Wif1 expression, and inhibition of the Wnt8/β-catenin pathway	Yao, X et al (57)
MSCs	siGARP	Lipofectamine transfection	Colon cancer	Inhibition of the proliferation, migration and invasion of colon cancer cells		Impeding IL-6 secretion and inactivating the JAK1/STAT3 pathway	Xing, H et al. (123)
UCMSCs	si-TGFβ1	Lipofectamine transfection	Lung cancer	Suppressing proliferation and increasing apoptosis in lung cancer cells		Inhibition of the Smad2/3, Akt/GSK-3β, MAPK, and NF-κB pathways	Zhao, X et al (124)
BMSCs	siGRP78	Lipofectamine transfection	Hepatocellular carcinoma	Inhibiting the growth and invasion of HCC cells and enhancing the sensitivity of HCC cells to sorafenib	Inhibition of the metastasis of sorafenib-resistant cells		Li, H et al (125)

BMSCs, bone marrow-derived mesenchymal stem cells; UCMSCs, umbilical cord-derived mesenchymal stem cells.

MSC-exosomes containing lncRNAs may represent a new option for treating tumors. However, there are still many challenges to be addressed. First, we need to have a deeper understanding of lncRNA function in tumorigenesis and development, and second, we need to choose suitable lncRNAs to apply to different types of tumor treatments. Finally, we need to explore whether different lncRNAs can be combined to improve the effectiveness of tumor treatment.

### 2.3 Effects of MSC-exosomes containing circRNAs on tumor treatment

CircRNAs are a new type of noncoding RNA that consists of covalently closed loops formed through backsplicing; these RNAs play key roles in cancer development and progression by acting as miRNA sponges and transcriptional regulators and combining with RNA binding proteins (131–133). In recent reports, the enrichment and stability of circRNAs in exosomes have been assessed (134). Overall, MSC-exosomes with circRNAs have potential applications as a new method of tumor treatment (**Table 3**). Yao X et al. found that circ_0030167 mainly regulated miR-338-5p, enhanced Wif1 expression, and inhibited the Wnt8/β-catenin pathway, thereby inhibiting pancreatic cancer cell stemness and tumor progression; MSC-exosomes could significantly inhibit the migration, invasion, proliferation, and stemness of pancreatic cancer cells by transferring circ_0030167 (57). However, the mechanism of circRNAs in tumor progression still needs to be explored. In general, MSC-exosomes containing circRNAs may represent a new tool for targeted tumor therapy.

### 2.4 Effects of exosomes derived from MSCs transfected with siRNAs on tumors

SiRNAs are a class of noncoding RNAs that are generally from 21 to 25 base pairs in length and are produced from longer dsRNA, which is cleaved by the dsRNA-specific endonuclease Dicer (135, 136). siRNAs have shown potential as a new therapeutic reagent in tumor treatment due to their gene-knockdown capability (137).

Because of their advantages in terms of homing and safety, MSC-exosomes have been considered the most promising carrier in tumor treatment. In recent reports, the effect of exosomes derived from MSCs transfected with siRNA in tumor treatment has been noted (**Table 3**). Xing H et al. found that GARP expression in exosomes derived from MSCs transfected with siGARP was significantly decreased and that siGARP-MSC-derived exosomes could inhibit the proliferation, migration and invasion of colon cancer cells by impeding IL-6 secretion and inactivating the JAK1/STAT3 pathway (123). Zhao X et al. found that MSC-exosomes could inhibit the proliferation and promote the apoptosis of lung cancer cells, yet MSC-exosomes could promote invasion and migration and induce EMT. Furthermore, exosomes derived from MSCs transfected with si-TGFβ1 enhanced the ability to suppress proliferation and increase apoptosis in lung cancer cells and eliminated the induction of EMT in lung cancer cells by inhibiting the Smad2/3, Akt/GSK-3β, MAPK, and NF-κB pathways (124). The above two studies show the ability of MSC-exosomes in tumor treatment, but the expression of genes that siRNAs knock down in tumors has not yet been justified. Exosomes derived from MSCs transfected with siGRP78 have been reported to target GRP78 and inhibit its expression in sorafenib-resistant hepatocellular carcinoma (HCC) cells, inhibit the growth and invasion of HCC cells and enhance the sensitivity of HCC cells to sorafenib (125).

Currently, the application of artificially modified MSC-exosomes in tumor treatment mainly focuses on preclinical studies and clinical trials are also gradually underway. Clinical trial for the treatment of solid tumors with artificially modified MSC-exosomes has been registered in the treatment of metastatic PDAC patients with KrasG12D mutation. M.D. Anderson Cancer Center sponsored a phase 1 clinical trial to explore the best dose and side effects of MSC-exosomes with the KrasG12D siRNA in treating participants with pancreatic cancer with KrasG12D mutation that has spread to other places in the body. Although the treatment results have not been published, we need to pay more attention and have more patience for the clinical transformation of artificially modified MSC-exosomes in tumor therapy. There are many issues, including GMP compliance, regulatory considerations, and safety profiles, that need to be solved before using exosomes in clinical practice. We believe that an increasing number of clinical trials will be applied to different types of solid tumors.

This new approach reveals the potential value of MSC-exosomes in tumor treatment, but researchers have paid attention to the effect of exosomes derived from MSCs transfected with siRNA on tumors, not the application of MSC-exosomes as carriers for tumor treatment. The potential of MSC-exosomes as therapeutic carriers for tumor treatment has not been fully exploited, and the application of MSC-exosomes modified directly with siRNAs in tumor treatment has not been studied. Therefore, this method may be a direction of future research.

## 3 MSC-exosomes as carriers of anticancer drugs for tumor treatment

A growing body of research suggests that MSCs are potential carriers for delivering anticancer drugs because of their ability to migrate specifically to tumors (138, 139). However, the tumor-promoting ability limits the application of MSCs in the clinical treatment of tumors. With the birth of nanotechnology in the 1990s, nanomedicine has become an important development direction of modern medicine for various disease therapeutics and diagnoses, and exosomes have emerged as promising nanomedicine carriers for tumor treatment due to their advantages (140, 141). It has been reported that MSC-exosomes can be internalized by tumor cells (142), and MSC-exosomes exhibit excellent characteristics in tumor treatment. Compared with traditional chemotherapy modalities, MSC-exosomes carrying anticancer agents can precisely deliver drugs into tumor cells to alleviate the side effects of drugs and greatly enhance tumor inhibition. Wei, H et al. found that MSC-exosomes carrying anticancer drugs could enhance cellular uptake in tumor cells but reduce cytotoxicity to normal cells and repair damaged tissues (34, 143, 144). Overall, MSC-exosomes can serve as carriers of anticancer drugs, bringing new ideas and hopes for the development of new tumor treatments.

### 3.1 Antitumor effects of MSC-exosomes modified directly with anticancer drugs

**Figure 2:**
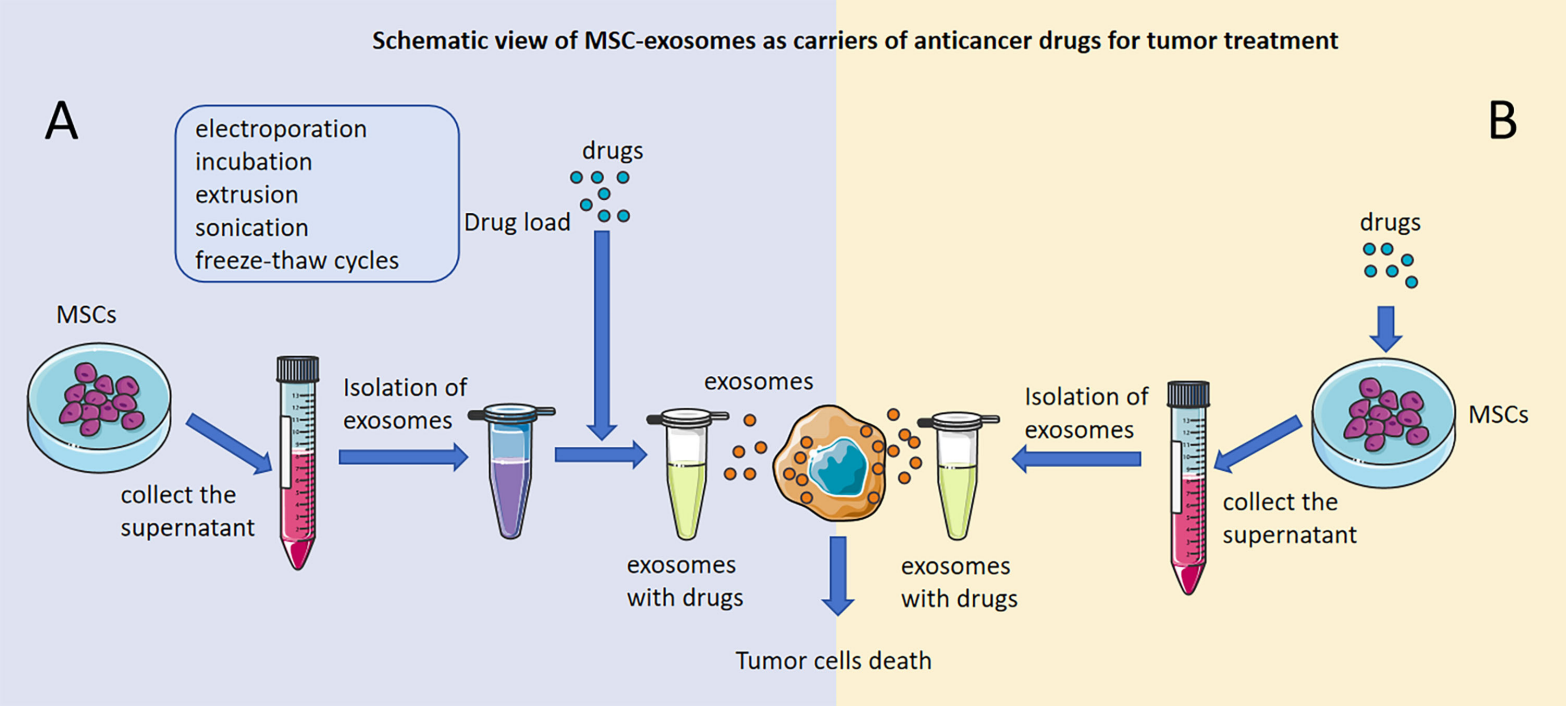
Schematic view of MSC-exosomes as carriers of anticancer drugs for tumor treatment. Antitumor effects of MSC-exosomes modified directly with anticancer drugs. **(A)** Antitumor effects of drug-loaded MSC-exosomes developed by direct modification of MSCs. **(B)** The figure was partly generated using Servier Medical Art, provided by Servier, licensed under a Creative Commons Attribution 3.0 unported license.

Generally, the preparation of MSC-exosomes with anticancer drugs involves two main approaches: direct and indirect modification. First, MSC-exosomes can be modified directly: we can collect MSC-exosomes from the cell supernatant of MSCs, and anticancer drugs can be directly loaded into MSC-exosomes by different loading methods, including electroporation (34), incubation (143), extrusion (145), sonication (146), and freeze−thaw cycles (145) (**Figure 2A**). Liang, L et al. loaded norcantharidin (NCTD) into MSCs to obtain MSC-exosomes with norcantharidin (MSC-exosome-NCTD) via electroporation, which exhibited positive loading efficiency, but pulses of electroporation may cause damage to anticancer drugs and the membrane integrity of MSC-exosomes. Compared with NCTD treatment alone, MSC-exosomes-NCTD treatment enhanced cellular uptake and apoptosis, induced HCC cell cycle arrest and inhibited HCC cell proliferation. MSC-exosomes-NCTD showed HCC tissue homing ability *in vivo* and repaired damaged liver tissues (34, 147, 148). Incubation is the most commonly used and easiest method to load drugs, but incubation exhibits low loading efficiency; sonication demonstrates better efficiency than incubation but can destroy MSC-exosome membranes and cause protein aggregation, impacting the efficiency of loading. Chen, M found that 5-fluorouracil (5-FU) could be loaded into MSC-exosomes via incubation and sonication and found that the sonication method had a higher loading efficiency than the incubation method. Moreover, MSC-exosomes-5-FU had good anti-cholangiocarcinoma efficacy and safety (146–148). Furthermore, doxorubicin (DOX) could be loaded into MSC-exosomes via electroporation and incubation. MSC-exosome-DOX exerted a stronger inhibitory effect than DOX alone on colon cancer and osteosarcoma and hindered the progression of tumors (33, 143, 144). In addition, extrusion and freeze−thaw cycles could also be applied as methods for loading anticancer drugs into MSC-exosomes, and uniformly sized MSC-exosomes with a high content of anticancer drugs could be obtained through extrusion, but damage to the MSC-exosome membrane during extrusion and freeze−thaw cycles limits the application of this approach in tumor treatment (145). In recent studies, electroporation and incubation have been the two main ways of loading anticancer drugs into MSC-exosomes directly.

### 3.2 Antitumor effects of drug-loaded MSC-exosomes developed by direct modification of MSCs

Indirect modification of MSC-exosomes has also been applied to load anticancer drugs, and we can collect the cell supernatant of MSCs that are incubated with antitumor drugs and then extract MSC-exosomes with antitumor drugs (**Figure 2B**). MSC-exosomes that have significant antitumor effects could be isolated from MSCs that were incubated with Taxol, vincristine and paclitaxel (PTX) (55, 56, 142). Pascucci L et al. found that MSC-exosomes could be isolated from MSCs incubated with PTX to form MSC-exosomes-PTX that release PTX into pancreatic cancer cells and have a significant antitumor effect. This was the first time that MSC-exosomes had been proven to be useful as carriers of anticancer agents to treat tumors, and this new strategy was presented as a way to precisely target tumors and release agents for tumor treatment (142). Vincristine could be loaded into MSCs directly via incubation, and then MSC-exosomes-vincristine could be isolated and could increase cytotoxicity for glioblastoma cells (56). However, the method of indirect modification of MSC-exosomes is seldom applied in practice and will be the direction of our future research.

Antitumor drugs have varying degrees of side effects on the human body including myocardial injury, nervous system injury and kidney damage. Reducing the impact of these side effects is a hot topic in tumor treatment for most researchers. Wei H et al. found that MSC-exosome-DOX could reduce the damage of DOX to the myocardium at the cellular and tissue levels (143, 144). Reducing the side effects of antitumor drugs is also our current research focus, and we conjecture whether a series of side effects can be alleviated by enhancing the targeting of MSC-exosomes to tumors.

In general, considering the unique advantages of MSC-exosomes in tumor treatment, MSC-exosomes can play a role as a vehicle for targeted drug delivery. How to improve the drug loading efficiency and the stability of MSC-exosome-antitumor drugs is our next research goal. We can combine two types of drug loading methods to increase drug loading efficiency, and we can utilize new materials such as hydrogels to improve the stability of drug loading systems.

## 4 MSC-exosomes as carriers of nanomaterials for tumor treatment

With the development of medicine, the combination of drug treatment and other modalities has become a new strategy for tumor treatment. MSC-exosomes are an emerging modality for the treatment of tumors, and their combination with other treatments will have an enormous impact on advancing the treatment of tumors. Due to their ability to home to tumors, MSC-exosomes exhibit a good ability to transport cargo. In recent studies, MSC-exosomes have been applied as tools to transfer new synthetic materials into tumor cells and have demonstrated great potential in tumor therapy. Altanerova, U et al. obtained MSC-exosomes containing Venofer from MSCs incubated with Venofer, an iron oxide magnetic nanoparticle, and found that MSC-exosomes containing Venofer could be efficiently internalized by tumor cells and facilitate targeted tumor cell ablation to achieve tumor therapeutic effects via magnetically induced hyperthermia (149). Furthermore, Rezaie Z et al. found that MSC-exosomes could be incorporated into polycaprolactone (PCL) nanofibers by electrospinning, and PCL-MSC-exosomes exhibited significant effects in inducing the apoptosis of breast cancer cells. PCL-MSC-exosomes could replace traditional antitumor drugs and become a new method of tumor treatment (54). The emerging modalities for the treatment of tumors have considerable advantages and reduce the limitations of traditional methods of tumor treatment, but the emerging modalities require many *in vivo* experiments to verify their safety. Currently, research on MSC-exosomes as carriers of nanomaterials for tumor treatment is still rare, but nanomedicine is in a rapid development stage, and a growing number of therapeutic nanomaterials are being developed for tumor treatment. We believe this method will be an attractive research hotspot. Faced with this emerging treatment model, we need to further evaluate the safety of nanomaterials considering their effect on protein stability and human metabolism and verify their safety using animal models.

## 5 Application of MSC-exosomes modified by genetic engineering in tumor treatment

After almost 30 years of development, genetic engineering has rapidly become a critical method of treating a variety of diseases, including blood disorders, genetic diseases and tumors (150–152). In particular, with continuous expansion and adaptation, genetic engineering has emerged as a method for the treatment of different types of tumors, and such strategies could enhance the efficacy and safety of tumor treatments as well as broaden the potential applications for tumor treatments. Furthermore, MSC-exosomes, as an emerging method in tumor treatment, could be combined with genetic engineering to dramatically alter the landscape of tumor treatment. Recent reports revealed that MSCs could be prepared via genetic engineering to express target genes that inhibit the progression of tumors. Engineered MSC-exosomes could also be isolated from engineered MSCs and applied to tumor treatment, which significantly improved the efficiency of tumor treatment (53, 153). Tumor necrosis factor-related apoptosis-inducing ligand (TRAIL) is a potential agent for cancer treatment, and a TRAIL/GFP encoding plasmid could be transfected into MSCs through PEI25Pyr50% (a nonviral vector) to obtain TRAIL-engineered MSCs. TRAIL-engineered MSC-exosomes demonstrated tumor-homing ability and inhibited tumor progression through abundant necrosis in melanoma cells in both *in vitro* and *in vivo* models (53). Altanerova U et al. used retrovirus infection to transfer the suicide fusion gene-yeast cytosine deaminase::uracil phosphoribosyl (yCD::UPRT) into MSCs, and yCD::UPRT-MSC-exosomes could be isolated from the conditioned medium (CM) of yCD::UPRT-MSCs and could be internalized by cancer cells. Moreover, yCD::UPRT-MSC-exosomes could convert the prodrug 5-fluorocytosine (5-FC) to 5-FU in cancer cells to efficiently induce tumor cell death (153). You, B et al. extracted MSC-exosomes with L-PGDS (MSC-exosomes- L-PGDS) from MSCs which transferred through adenovirus encoding L-PGDS, and found that MSC-exosomes- L-PGDS could inhibit the colony-forming, migration, and invasion abilities of gastric cancer cells *in vitro* and inhibit the tumor growth in a nude mouse subcutaneous tumor-bearing model *in vivo (* 154). Genetically engineered MSC-exosomes have been considered a promising tumor treatment strategy, but studies on their application are still in their infancy, and further research is needed. The development of more effective and safer genetic engineering therapy methods for MSCs remains a challenge in tumor treatment.

## 6 Discussion

As a cell-free system, MSC-exosomes can be employed in various promising approaches for tumor treatment that exhibit safety because of their limited effect on normal cell viability. MSC-exosomes have been used in the clinical treatment of different diseases, including steroid-refractory graft-versus-host disease (155), chronic kidney disease (156), and liver diseases (157), polycystic ovary syndrome (158). However, there are still a few concerns that need to be resolved before applying MSC-derived exosomes in tumor clinical therapy. First, to achieve the large-scale preparation of exosomes, the optimization of methods for isolating exosomes from MSCs is the most important step. At present, the main methods for isolating exosomes include sequential ultracentrifugation, gradient ultracentrifugation, ultrafiltration, size-exclusion chromatography, polymer precipitation, immunoaffinity capture and microfluidics-based techniques. In recent years, although great progress has been made in exosome isolation methods, many problems still need to be solved, such as contamination, low purity, high cost, and low yields (159). The combination of two different types of isolation methods may yield a rapid and cost-effective method for exosome isolation. Concurrently, the number of exosomes produced by cells could be increased. Faezeh Vakhshiteh F et al. found that the abundance of exosomes is related to the source of MSCs. Due to the high proliferation rate and the great capacity of pulp-derived MSCs (DPSCs) to differentiate into different cells, dental pulp has the ability to secrete more exosomes, and DPSCs could be suggested as the ideal cell type for high-yield exosome production (52). Overall, to apply MSC-exosomes for tumor treatment in the clinic, exosome isolation technology should be improved and developed to enable the isolation of high concentrations of exosomes with high efficiency, and suitable sources for isolating MSCs should be assessed. Moreover, the antitumor effect of MSC-exosomes is related to the source of MSCs, processing mode and type of cancer (160). Del Fattore A et al. found that the effects of BMSC-exosomes UCMSC-exosomes and ATMSC-exosomes on the proliferation and apoptosis of glioblastoma cells are different, and different types of MSC-exosomes have various influences on the same tumor (56). In addition to the source of MSCs, exosomes from MSCs treated in different ways exert different effects on tumor cells. Zhang X et al. found that hypoxic MSC-exosomes could promote the invasion of lung cancer cells and EMT by activating STAT3 signaling via the aberrant expression of miRNAs (miR-193a-3p, miR-210-3p and miR-5100) in hypoxic MSC-exosomes (161). Overall, patients with different tumors have different characteristics, and choosing the appropriate MSC source and processing mode is the key to improving the treatment effect.

Notably, the inhibitory effect of MSC-exosomes on tumor progression has been indicated by an increasing number of studies. It has been reported that MSC-exosomes can enhance the cytotoxicity of radiotherapy and chemotherapy in tumors and metastatic tumor foci (1, 39). Combining MSC-exosomes with other treatments, such as radiotherapy and chemotherapy, could greatly improve the efficiency of tumor treatment. Furthermore, MSC-exosomes could be used in tumor treatment directly because MSC-exosomes are rich in miRNAs that could inhibit the progression of tumors. Lee JK et al. found that miR-16 was enriched in MSC-exosomes and could inhibit angiogenesis *in vitro* and *in vivo* by significantly downregulating vascular endothelial growth factor (VEGF) in breast cancer cells, and Pakravan K et al. found that MSC-exosomes were rich in miR-100 and could suppress the angiogenesis of breast cancer by modulating the mTOR/HIF-1α/VEGF signaling axis (40, 41). To our surprise, some studies have found that MSC-exosomes exhibit great potential in the treatment of leukemia; it has been reported that MSC-exosomes are rich in miR-124-5p and miR-222-3p and suppress the progression of acute myeloid leukemia by downregulating SMC4C and targeting IRF2/INPP4B (42, 43). The advantage of this characteristic of MSC-exosomes gives us a new idea to explore the potential of MSC-exosomes in tumor treatment. However, there is growing evidence indicating that MSC-exosomes can promote tumor growth (44–46), metastasis (45, 47), immunosuppression (48) drug resistance (49) and dormancy in cancer cells (50). Scientists have recently reported that MSC-exosomes could promote the growth of gastric cancer, colon cancer and osteosarcoma (44–46) and enhance the migration of breast cancer cells and gastric cancer cells (45, 47). Recent reports have shown that immunosuppression is an essential factor affecting tumor development. Wang J et al. found that MSC-derived exosomes could promote the release of NO from myeloid-derived suppressor cells to enhance their inhibitory effects on T cells through the STAT1 and STAT3 pathways to induce immunosuppression and promote the progression of multiple myeloma (48). MSC-exosomes can also activate the CaM-Ks/Raf/MEK/ERK pathway to induce drug resistance in gastric cancer cells, and MSC-exosomes can promote osteosarcoma and gastric cancer cell growth through activation of the hedgehog signaling pathway (45, 46). Deng, M et al. found that lnc00461 is highly expressed in patients with multiple myeloma and that lnc00461 can be transferred into multiple myeloma cells through MSC-derived exosomes and promote multiple myeloma tumorigenesis by regulating miR-15a/16 and BCL-2 (162). It has been reported that miR-23b is enriched in MSC-exosomes and can promote dormancy in metastatic breast cancer cells by decreasing MARCKS expression (50). To address the limitation of the tumor-promoting effect, we chose to artificially modify MSC-exosomes in tumor treatment. Through current research, we determined that artificially modified MSC-exosomes could serve as a potential selection for tumor treatment in future research.

Improving treatment effectiveness is an important indicator of tumor treatment methods. We propose that engineering treatment with MSC-exosomes can improve tumor targeting to improve the therapeutic effect, and we could load modified targeted peptides onto MSC-exosomes membranes to improve tumor targeting. Furthermore, the safety of MSC-exosomes in the treatment of tumors is also an important indicator for preclinical application, although artificially modified MSC-exosomes could significantly reduce the promoting effect of MSC-exosomes on tumor progression, but this not enough. MSC-exosomes mainly exert antitumor effects through contents consisting of miRNAs, proteins, lipids and metabolites, which will be the focus of future research. How to use this characteristic in tumor treatment is worth considering (37). Furthermore, the content of MSC-exosomes also had promoted tumors. To eliminate their impact on tumor progression, whether we could process MSC-exosomes before application to clean up the contents of the MSC-exosomes, we conjectured that reformation of the MSC-exosomes membrane upon sonication resulted in clearance of MSC-exosomes contents. Therefore, we could improve the safety of MSCs-exosomes therapy via this method.

## 7 Conclusions and perspectives

We highlighted the great potential of MSC-exosomes in tumor treatment compared with traditional methods of tumor treatment, but the dual effects of exosomes from MSCs on tumors limit their application. We propose the feasibility of using artificially modified MSC-exosomes to treat tumors. There are still considerable challenges regarding the preparation of high-concentration exosomes with high efficiency and the feasibility of clinical application. While studies on the effects of artificially modified MSC-exosomes in tumor treatment are still in the early development period, research progress in the field provides hope that artificially modified MSC-exosomes could be regarded as an efficient, safe and targeted therapy for tumors with minor side effects.

## Author contributions

YS: Writing – original draft, Writing – review & editing. QS: Writing – review & editing. DH: Writing – review & editing. BS: Writing – review & editing. MG: Writing – review & editing. XL: Writing – review & editing. BQ: Writing – review & editing. LS: Writing – review & editing. ZY: Writing – original draft, Writing – review & editing. LW: Writing – original draft, Writing – review & editing.
